# Awareness and Knowledge of Hepatitis B Vaccination Among Newly Enrolled First-Year Medical Undergraduates in South India: A Cross-Sectional Survey

**DOI:** 10.7759/cureus.73567

**Published:** 2024-11-13

**Authors:** Rudramurthy K G, Kanyakumari D H, Naveen Kumar Madalageri, Harish Rangareddy

**Affiliations:** 1 Microbiology, Haveri Institute of Medical Sciences, Haveri, IND; 2 Physiology, Haveri Institute of Medical Sciences, Haveri, IND; 3 Pharmacology, Haveri Institute of Medical Sciences, Haveri, IND; 4 Biochemistry, Haveri Institute of Medical Sciences, Haveri, IND

**Keywords:** health education and awareness, hepatitis b vaccine, knowledge mapping, medical student, risk

## Abstract

Background: Hepatitis B poses a significant public health risk, particularly for healthcare professionals who face heightened exposure in clinical settings. This study assesses the awareness and knowledge of hepatitis B vaccination among first-year medical undergraduates in their preclinical stage before transitioning to clinical phases.

Methods: This cross-sectional survey was conducted among newly enrolled first-year medical undergraduate students at a medical college before the commencement of a planned vaccination drive, allowing the institution to assess baseline knowledge and logistical needs for the drive. A self-administered questionnaire was used to evaluate students' awareness and understanding of hepatitis B transmission, vaccination, and the occupational risks associated with healthcare work. The data gathered from this survey provided critical insights for optimizing the logistics and educational components of the upcoming vaccination program for the incoming cohort.

Results: Among the 126 respondents, there was a slightly higher representation of male participants (53.17%, n=67), while 46.83% (n=59) were females. Awareness of hepatitis B as a highly contagious liver infection was high, with 88.9% of students agreeing or strongly agreeing. The knowledge that hepatitis B is a public health concern and can lead to severe conditions such as liver cancer was similarly prevalent (97.6% and 90.4%, respectively). Most respondents (80.2%) were aware of the heightened risk for healthcare professionals and viewed vaccination as an effective preventive measure (96.1%). Awareness of transmission routes, including contact with infected blood (96%) and unprotected sexual contact (97.6%), was also strong. However, fewer students were informed about the recommended vaccination schedule (63.4%) or the necessity of booster doses for high-risk individuals (70.6%). Additionally, 93.7% believed healthcare workers should be vaccinated, while 88.9% recognized the need for immunity status checks in healthcare settings. Only 16.66% (n=21) reported receiving the hepatitis B vaccine, with 14.28% having completed the full three-dose series, while 2.38% received only one or two doses. A significant portion (62.69%) reported not being vaccinated, and 20.63% were unsure of their vaccination status. Key reasons for incomplete vaccination included lack of awareness about the full series (58.73%), fear of side effects (6.34%), and perceived lack of necessity (7.93%). Access challenges were also noted by 2.38% of students. Only 1.58% of respondents had checked their immunity status through anti-hepatitis B surface antibody (HBsAb) testing.

Conclusion: Newly enrolled medical undergraduates show substantial foundational knowledge of hepatitis B and its prevention. However, targeted educational and peer-led initiatives are recommended to bridge the remaining gaps, ensuring future healthcare professionals have comprehensive knowledge for effective hepatitis B prevention. The findings also underscore the need for improved vaccination awareness and accessibility to achieve comprehensive immunization among medical undergraduates.

## Introduction

Hepatitis B is a liver-targeting viral infection capable of causing both acute and chronic disease. The virus is primarily transmitted from mother to child during childbirth, in early childhood, and through contact with infected blood or bodily fluids. Transmission can occur during sexual activity with an infected individual, unsafe injections, or exposure to contaminated sharp instruments [1]. According to the World Health Organization (WHO), an estimated 254 million people were living with chronic hepatitis B infection in 2022, with approximately 1.2 million new infections annually. In that same year, hepatitis B was responsible for around 1.1 million deaths, primarily due to complications such as cirrhosis and hepatocellular carcinoma (a form of primary liver cancer) [1].

Medical students are at increased risk of hepatitis B infection due to potential exposure to blood and body fluids during cadaveric dissection, clinical rotations, and autopsy postings. Although hepatitis B is preventable with safe and effective vaccines, gaps in awareness and knowledge among medical students remain concerning [2].

Many preclinical students are aware of hepatitis B and its primary transmission routes, substantial gaps in hepatitis B virus (HBV) knowledge, vaccination compliance, and awareness of clinical features and complications persist [3]. Various studies suggest that only a small fraction of medical students are fully vaccinated or aware of their immunization status [2-4]. Therefore, implementing preclinical vaccine checks alongside an infectious disease awareness and prevention program is essential to enhance hepatitis B knowledge and protection among medical students.

In India, hepatitis B vaccination is generally mandatory for first-year medical (MBBS) students, with most medical colleges enforcing it as a pre-admission health requirement or during the early years of the course. This survey, therefore, focuses on assessing the awareness and knowledge of the newly enrolled first-year students, aiming to identify informational gaps that, once addressed, could ensure the successful vaccination and preparedness of students as they advance in their medical education.

## Materials and methods

Study design

This cross-sectional survey was conducted among first-year medical undergraduate (MBBS) students at a medical college who had recently enrolled in the program for the academic year 2024-2025 at Haveri Institute of Medical Sciences, Haveri, in South India. The study was carried out in the month of October 2024.

Study setting

The study was conducted at Haveri Institute of Medical Sciences before the vaccination drive for first-year MBBS students to perform the needs assessment and plan the logistics.

Participants and sampling

Students were briefed in person by the researchers about the study's purpose and procedures. All first-year students were included through universal sampling. The sample size (n) for the survey was calculated with the following equations: x = Z (c/100)2r (100-r) and n = N x/((N-1) E2+x), where N is the population size, E is the margin of error (10%), r is the fraction of responses (50%), and Z (c/100) is the critical value for the confidence level c (5%). The first MBBS student population in Karnataka at the time of the study was 11,745 [5]. A margin of error of 10% was initially assumed, and the sample size obtained was 96. It was decided to proceed with purposive sampling of only newly enrolled first-year MBBS students at the time of the study.

Data collection tool

A study questionnaire was developed by the authors (RKG and KDH) and was used for data collection (Appendices). To validate the content, the initial version of the questionnaire was reviewed by a panel of subject matter experts, including faculty members from the Departments of Microbiology, Physiology, and Pharmacology. Their feedback helped refine the questions for clarity, relevance, and comprehensiveness. The reliability of the questionnaire administered in this study was calculated, yielding a coefficient of 0.9. While this value indicates high internal consistency and reliability, it is important to clarify that this initial questionnaire was specifically designed to assess knowledge regarding hepatitis B vaccination among newly enrolled first-year MBBS students. Given its exploratory nature, we prioritized gathering preliminary insights into the participants' awareness.

Before participating, students were thoroughly informed about the study's objectives, methodology, and the nature of the questionnaire. The importance of providing honest and thoughtful responses was emphasized to ensure informed participation and enhance the validity of the feedback collected.

Ethical clearance for the study was obtained from the Institutional Ethics Committee of Haveri Institute of Medical Sciences (reference number: HIMSHVR/ADMN/IEC/002/2024-25). The cross-sectional descriptive study employed a purposive sampling methodology, with a total of 126 students participating. Second-year students were excluded, as they had already completed the vaccination and were more knowledgeable about hepatitis B vaccination due to their clinical postings and microbiology training.

To prevent bias, the researchers involved in subject selection did not partake in administering the questionnaire or collecting data. A separate team handled these tasks to ensure impartiality. The questionnaire was distributed using Google Forms (Google, Inc., Mountain View, CA) to assess hepatitis B vaccination awareness and knowledge among first-year MBBS students. The form was configured to allow only one response per participant, ensuring data integrity by preventing multiple submissions.

Before accessing the questionnaire, participants were presented with a consent statement on the first page of the form. Only after clicking the consent box to indicate their agreement to participate in the study could they proceed to the next section of the survey. This ensured that all participants provided explicit informed consent before responding to the questions. Participation in the survey was voluntary, and all responses were kept confidential. No identifying information, such as names or student IDs, was collected, ensuring anonymity. The data was securely stored in a password-protected format, accessible only to the research team. Confidentiality was maintained throughout the study, and the results were used solely for research purposes to evaluate hepatitis B vaccination awareness among medical students.

Data analysis

The number and percentage of students responding to each item were recorded, and proportions were calculated using Microsoft Excel (Microsoft Corp., Redmond, WA). Statistical analysis was performed using IBM SPSS Statistics for Windows version 16 (SPSS Inc., Chicago, Ill), as well as for calculating Cronbach's alpha and descriptive statistics. The data was summarized, with results presented as percentages, and any identified knowledge gaps were discussed.

## Results

A total of 126 recently enrolled first-year MBBS students took part in the survey, offering valuable insights into their awareness and comprehension of hepatitis B vaccination. Below, we present the demographic characteristics of the participants along with their responses to the questionnaire.

Demographics

The demographic profile of the 126 students who participated in the study revealed that the majority of the students have a wide age range from 17 to 24 years. The participants were predominantly aged 19 years, which constituted 44.44% (n=56) of the sample. The age distribution revealed that 26.98% (n=34) of the participants were 18 years old, 14.29% (n=18) were 20 years old, and 7.94% (n=10) were 21 years old. Participants aged 17 (n=2, 1.58%), 22 (n=4, 3.17%), 23 (n=1, 0.79%), and 24 (n=1, 0.79%) years accounted for smaller proportions of the sample, ranging from 0.79% to 3.17%, reflecting a young adult cohort typical of first-year medical students. In terms of gender, there was a slightly higher representation of male participants, with 53.17% (n=67) identifying as male and 46.83% (n=59) as female. The demographic data suggest a relatively balanced distribution of age and gender within the study cohort, with a slight male predominance as shown in Table 1.

**Table 1:** Age groups of the first-year MBBS students and overall gender characteristics

Age group (years)	Number of participants	Percentage (%)
17	2	1.59%
18	34	26.98%
19	56	44.44%
20	18	14.29%
21	10	7.94%
22	4	3.17%
23	1	0.79%
24	1	0.79%
Gender	Number of participants	Percentage (%)
Male	67	53.17%
Female	59	46.83%

A total of 126 respondents participated in the survey assessing awareness and perceptions regarding hepatitis B among healthcare students. The results depicted in Table 2 reveal a high level of awareness of hepatitis B as a contagious liver infection, with 88.9% agreeing or strongly agreeing (47.6% agree and 41.3% strongly agree) to the statement. Additionally, 97.6% recognized hepatitis B as a major global public health concern, with a majority agreeing (57.9%) or strongly agreeing (39.7%).

**Table 2:** Awareness and knowledge regarding hepatitis B among the newly enrolled first-year medical undergraduates

Question	Statement	Response options	Number of respondents (n=126)	Percentage (%)
Q1	I am aware that hepatitis B is a highly contagious liver infection.	Strongly agree	52	41.3%
		Agree	60	47.6%
		Neutral	10	7.9%
		Disagree	1	0.8%
		Strongly disagree	3	2.4%
Q2	I know that hepatitis B is a major public health concern worldwide.	Strongly agree	50	39.7%
		Agree	73	57.9%
		Neutral	3	2.4%
Q3	I am informed that hepatitis B can lead to serious health conditions, such as liver cancer or cirrhosis.	Strongly agree	56	44.4%
		Agree	58	46.0%
		Neutral	12	9.5%
Q4	I am aware that healthcare professionals are at an increased risk of contracting hepatitis B.	Strongly agree	36	28.6%
		Agree	65	51.6%
		Neutral	19	15.1%
		Disagree	5	4.0%
		Strongly disagree	1	0.8%
Q5	I understand that vaccination is an effective preventive measure against hepatitis B.	Strongly agree	66	52.4%
		Agree	55	43.7%
		Neutral	4	3.2%
		Disagree	1	0.8%
Q6	I know that hepatitis B can be transmitted through contact with infected blood or body fluids.	Strongly agree	72	57.1%
		Agree	49	38.9%
		Neutral	5	4.0%
Q7	I am aware that sharing needles or syringes can transmit hepatitis B.	Strongly agree	81	64.3%
		Agree	44	34.9%
		Neutral	1	0.8%
Q8	I know that unprotected sexual contact can increase the risk of hepatitis B infection.	Strongly agree	75	59.5%
		Agree	48	38.1%
		Neutral	3	2.4%
Q9	I know that hepatitis B can be passed from an infected mother to her child at birth.	Strongly agree	63	50.0%
		Agree	53	42.1%
		Neutral	10	7.9%
Q10	I know that casual contact, like shaking hands, does not spread hepatitis B.	Strongly agree	75	59.5%
		Agree	43	34.1%
		Neutral	3	2.4%
		Disagree	1	0.8%
Q11	I know that the hepatitis B vaccine is safe and has few side effects.	Strongly agree	27	21.4%
		Agree	73	57.9%
		Neutral	25	19.8%
		Disagree	1	0.8%
Q12	I am aware that the hepatitis B vaccine is effective in preventing infection.	Strongly agree	40	31.7%
		Agree	74	58.7%
		Neutral	10	7.9%
		Disagree	2	1.6%
Q13	I understand that the vaccine offers long-term protection against hepatitis B.	Strongly agree	40	31.7%
		Agree	68	54.0%
		Neutral	16	12.7%
		Disagree	2	1.6%
Q14	I believe that healthcare professionals should be vaccinated against hepatitis B.	Strongly agree	66	52.4%
		Agree	51	40.5%
		Neutral	6	4.8%
		Disagree	2	1.6%
		Strongly disagree	1	0.8%
Q15	I know that completing the full vaccination series is necessary for effective immunity.	Strongly agree	60	47.6%
		Agree	61	48.4%
		Neutral	5	4.0%
Q16	I am aware of the recommended hepatitis B vaccination schedule.	Strongly agree	24	19.0%
		Agree	56	44.4%
		Neutral	38	30.2%
		Disagree	7	5.6%
		Strongly disagree	1	0.8%
Q17	I am informed that booster doses may be recommended for high-risk individuals.	Strongly agree	27	21.4%
		Agree	62	49.2%
		Neutral	33	26.2%
		Disagree	3	2.4%
		Strongly disagree	1	0.8%
Q18	I believe that all healthcare workers should check their hepatitis B immunity status regularly.	Strongly agree	43	34.1%
		Agree	69	54.8%
		Neutral	14	11.1%
Q19	I understand that hepatitis B vaccination is the key to reducing the virus spread in healthcare settings.	Strongly agree	55	43.7%
		Agree	66	52.4%
		Neutral	5	4.0%

Awareness of severe health consequences associated with hepatitis B, such as liver cancer or cirrhosis, was also high, with 90.4% responding in agreement or strong agreement. Respondents largely understood the elevated risk faced by healthcare professionals, as 80.2% agreed or strongly agreed that they are at an increased risk of infection, although a small percentage expressed neutrality (15.1%) or disagreement (4.8%).

There was strong consensus regarding vaccination as an effective preventive measure, with 96.1% agreeing or strongly agreeing on its efficacy. Knowledge of hepatitis B transmission routes was notably high, with 96% of respondents aware that the virus could be transmitted through blood or body fluids and 99.2% recognizing shared needles as a transmission risk. Similarly, awareness of sexual transmission was evident, with 97.6% indicating agreement. However, only 50% of respondents expressed strong agreement that hepatitis B could be passed from mother to child, with 42.1% agreeing and 7.9% neutral.

Knowledge about non-transmission through casual contact was affirmed by 93.6% of respondents, highlighting a strong understanding of transmission specifics. In terms of vaccine safety, 79.3% agreed or strongly agreed that the hepatitis B vaccine is safe and has minimal side effects. Additionally, 90.4% were aware of the vaccine's effectiveness in preventing infection, with only 1.6% disagreeing.

The necessity of completing the full vaccination series for effective immunity was recognized by 96%, while awareness of the recommended vaccination schedule showed a wider distribution, with 63.4% in agreement or strong agreement but 30.2% remaining neutral. Knowledge regarding booster doses for high-risk individuals was acknowledged by 70.6%, although a notable portion (26.2%) was neutral on this topic. Overall, there was a strong endorsement (95.2%) for vaccination of healthcare professionals, with a high level of agreement on the need for regular immunity checks (88.9%) and the importance of vaccination to limit hepatitis B spread in healthcare settings (96.1%).

Table 3 summarizes the proportion of students who have received any doses of the vaccine, completed the full three-dose series, and have yet to begin vaccination. It also highlights common reasons for incomplete vaccination, such as lack of awareness about the series requirement and fear of side effects, along with data on the frequency of immunity checks (anti-hepatitis B surface antibody (HBsAb) testing).

**Table 3:** Hepatitis B vaccination status among the newly enrolled first-year medical undergraduates HBsAb: hepatitis B surface antibody

Vaccination status	Number (%)
Have you received the hepatitis B vaccine?	
Yes	21 (16.66%)
No	79 (62.69%)
Not sure	26 (20.63%)
If yes, have you completed the full hepatitis B vaccination series (3 doses)?	
Yes, completed all doses	18 (14.28%)
No, received only one or two doses	3 (2.38%)
If not completed, reasons for not completing the series or not taking the vaccine	
Difficulty accessing the vaccine	3 (2.38%)
Fear of side effects	8 (6.34%)
Perception that the vaccine is unnecessary	10 (7.93%)
Lack of awareness about the full vaccination series	74 (58.73%)
Others	31 (24.60%)
When was the last dose of the hepatitis B vaccine received?	
Within the last 6 months	3 (2.38%)
6 months to 1 year ago	1 (0.80%)
More than 1 year ago	17 (13.49%)
Not applicable	105 (83.33%)
Have you ever checked your immunity status (anti-HBsAb levels) for hepatitis B?	
Yes	2 (1.58%)
No	0 (0)
Not sure	19 (15.08%)

## Discussion

Our study reveals strong baseline awareness and knowledge of hepatitis B and its preventive measures among the first-year MBBS students who had recently enrolled in the MBBS program. The majority of participants understood hepatitis B transmission routes, the occupational risks for healthcare professionals, and the importance of completing the vaccination series. These findings suggest that educational efforts at this early stage may foster safer practices as students enter clinical rotations.

Our results align closely with those of Bhattarai et al., who also found high awareness levels among medical students regarding hepatitis B vaccination, coupled with comparable rates of vaccine completion [6]. Unlike our preclinical cohort, the participants in the study by Bhattarai et al., who had begun clinical rotations, experienced higher exposure risks, with 42.8% reporting sharps injuries from needles [6]. Although our students have not yet been exposed to such risks, the high level of hepatitis B awareness indicates preparedness and understanding of future occupational hazards.

In contrast, Asif et al. observed lower vaccination rates in a similar population, with only 57% vaccinated and 87.8% completing the full series [7]. This discrepancy may stem from variations in institutional policies and access to vaccination programs. Their study also highlighted motivational barriers to vaccination, such as low perceived risk and fear of injections, underscoring the need for ongoing education to address these psychological barriers [7].

Studies in other South Asian regions, such as that of Shrestha et al. in Nepal, reported even lower vaccination completion rates, with only 37% of preclinical students fully vaccinated [8]. Their findings also support the need for early vaccination, a concept reflected in our high compliance rates among students who have entered the second year (129 out of 149, 89.6%, unpublished data), who have completed the vaccination series through our institutional vaccination drive. This suggests that the availability of free vaccination programs may play a critical role in compliance.

Comparatively, Butt et al. reported that only 33% of clinical-year medical students in Islamabad were fully vaccinated, much lower than the rate observed in our study [9]. This may reflect differences in institutional policies and vaccine accessibility. Bajaj et al. similarly found lower vaccination rates among Indian medical students (59%), emphasizing that lack of motivation and vaccine hesitancy are significant barriers to compliance [10]. Our study suggests that addressing these psychological factors through targeted interventions could improve vaccination rates among medical students, as supported by studies on healthcare students' attitudes toward vaccinations [7,9,10].

Similar challenges in compliance are seen globally. Okeke et al. in Nigeria attributed lower vaccination rates to limited institutional access, with non-compliance largely due to the lack of vaccine availability [11]. Our institution's vaccination policy likely contributed to the higher completion rate among second-year students, highlighting the importance of accessible vaccination programs in medical education. Likewise, Nasir et al. reported only a 42.2% vaccination rate, largely due to low perceived risk and high costs in low-income settings, where institutions often do not provide vaccination support [12].

A study by Alhowaish et al. showed high awareness levels of hepatitis B transmission routes among medical students, similar to our cohort's understanding. However, only 38% of the participants in their study completed the vaccination series, further demonstrating the need for a positive impact of vaccine accessibility on compliance rates [13]. These findings emphasize the importance of institutional support in ensuring vaccination completion.

In Sub-Saharan Africa, Aroke et al. found that although 83% of medical students in Cameroon were knowledgeable about hepatitis B, only 16.8% were fully vaccinated, reflecting significant cultural and psychological barriers, such as fear of side effects [14]. Similarly, Bajaj et al. in India identified vaccine hesitancy and lack of motivation as significant obstacles, underscoring the need for interventions targeting vaccine acceptance [10].

In the study by Abdela et al. in Nigeria, only 2% of medical students completed the three-dose series despite high knowledge of infection risk and prevention measures [15]. This low uptake, coupled with high exposure risk from needlestick injuries, suggests that educational institutions should not only encourage vaccination but also promote safe clinical practices.

Furthermore, Rathi et al. reported that only 8% of medical students had completed the vaccination series, with an alarming 73.3% never immunized despite the high-risk healthcare environment [16]. These findings highlight a critical need for educational institutions to play an active role in ensuring early vaccination to reduce occupational hepatitis B virus (HBV) exposure.

Our study also found that while institutional sources were the primary source of hepatitis B information, many students relied on informal sources such as family and media. This trend is consistent with studies by Thaver and Kamal [17] and Alam et al. [18], who reported that young adults often depend on informal channels for health-related information. This finding highlights the need for educational programs to provide clear, evidence-based information directly from healthcare providers.

Approximately two-thirds of the study participants reported that they had not received any doses of the hepatitis B vaccine, while about one-third had either completed the full three-dose series or had initiated vaccination without completing it. This partial vaccination status, combined with the number of unvaccinated individuals, indicates that while knowledge about hepatitis B is prevalent, it does not directly translate into full vaccination adherence. This aligns with findings from Bhattarai et al., who also reported high awareness but noted that many students still encountered gaps in vaccination completion despite being at risk of occupational exposure [6].

Among those who had begun the vaccination process but had not completed it, several barriers emerged. The primary reason cited was a lack of awareness regarding the requirement for a complete three-dose series. Accessibility issues also contributed to partial completion, with participants noting difficulty accessing vaccines, as well as perceived barriers such as cost and vaccine availability. In addition, fear of side effects deterred some students, underscoring the psychological factors that may inhibit full vaccination. Some participants also expressed a belief that vaccination was unnecessary, reflecting a gap in risk perception and perceived need, which is consistent with findings in other studies where students' perceived low risk contributed to low vaccination rates [7,10].

Comparative studies have similarly highlighted barriers affecting hepatitis B vaccine adherence in various settings. Asif et al. found lower vaccination rates in their study, where institutional barriers and personal reluctance due to low perceived risk affected compliance [7]. This trend is also observed in other low-resource settings; Shrestha et al. in Nepal reported only 37% completion among preclinical students, where institutional support may be lacking [8].

The substantial number of "not sure" responses from students regarding both their vaccination status and immunity testing highlights gaps in guidance and record-keeping about vaccination history. This may suggest a need for structured protocols in medical institutions to keep students informed about their vaccination records and antibody status. Given the importance of hepatitis B immunity in healthcare settings, especially among trainees, implementing protocols for immunity checks could help ensure comprehensive protection and promote regular updates on immunity status.

The observed gaps in vaccination rates and series completion reflect a broader pattern seen globally. Butt et al. in Islamabad reported only 33% of clinical-year students were fully vaccinated, lower than our findings, suggesting that institutions need to take proactive steps to promote and facilitate vaccination access [9]. Similarly, Okeke et al. in Nigeria attributed non-compliance largely to vaccine unavailability and low institutional support, which parallels our findings of accessibility challenges [11]. Moreover, the presence of psychological barriers, such as vaccine hesitancy and fear of side effects, has been well-documented in other studies. Bajaj et al. identified vaccine hesitancy as a significant barrier among Indian medical students, underscoring the need for interventions targeting vaccination acceptance and risk awareness [10]. The study by Nasir et al. in low-resource settings where costs posed a substantial barrier highlights the critical role that institutional support, in the form of accessible vaccination programs, plays in achieving high vaccination coverage [12].

Most participants who completed the vaccination series reported having received their last dose over a year ago, with only a minority receiving doses within the past six months. This suggests that while a proportion of students have initiated vaccination recently, a significant number may have received prior doses as part of an earlier healthcare protocol. Furthermore, the majority of participants had not checked their immunity status (anti-HBsAb levels), even among those who had completed the series, which may reflect a lack of emphasis on post-vaccination immunity assessment in the community setting. A small subset of students, primarily those who completed the series, reported antibody testing, possibly due to increased healthcare awareness or prior medical guidance.

Limitations of the study

Lack of HBsAb Titer Verification

This study did not measure anti-hepatitis B surface antibody (HBsAb) titers to confirm participants' vaccination status, which could affect the accuracy of reported compliance.

Potential Biases

Of the students, 20.63% were not sure of their vaccination status, indicating that documentation through electronic medical records should facilitate the proper documentation of vaccination.

Cross-Sectional Design

The cross-sectional approach limits the ability to infer causation between awareness and knowledge, attitudes, and vaccination practices, suggesting a need for longitudinal studies to track changes over time.

Focus on Awareness and Knowledge

This study assessed only awareness and knowledge, without evaluating students' behaviors or adherence to preventive practices. Students' actual preventive behaviors, which are essential for effective hepatitis B prevention, shall be assessed subsequently as the students advance into clinical settings.

## Conclusions

In conclusion, while our study reveals high awareness levels of hepatitis B among the newly enrolled first-year medical students, the relatively low completion rate or the lack of documentation of the vaccine series indicates the need for institutional interventions to address gaps in vaccination and immunity checks. The findings from this study point to several areas for improvement in hepatitis B vaccination efforts not only within medical education but also in the community. The current medical education curriculum focusing on the mandatory completion of the full three-dose series, along with the role of immunity testing, will ensure the vaccination of all medical students as they transition to clinical settings.

## Appendices

The study questionnaire developed and used for data collection is shown in Table 4.

**Table 4:** Self-administered questionnaire

Question		Statement	Response options
Q1		I am aware that hepatitis B is a highly contagious liver infection.	Strongly agree
			Agree
			Neutral
			Disagree
			Strongly disagree
Q2		I know that hepatitis B is a major public health concern worldwide.	Strongly agree
			Agree
			Neutral
Q3		I am informed that hepatitis B can lead to serious health conditions, such as liver cancer or cirrhosis.	Strongly agree
			Agree
			Neutral
Q4		I am aware that healthcare professionals are at an increased risk of contracting hepatitis B.	Strongly agree
			Agree
			Neutral
			Disagree
			Strongly disagree
Q5		I understand that vaccination is an effective preventive measure against hepatitis B.	Strongly agree
			Agree
			Neutral
			Disagree
Q6		I know that hepatitis B can be transmitted through contact with infected blood or body fluids.	Strongly agree
			Agree
			Neutral
Q7		I am aware that sharing needles or syringes can transmit hepatitis B.	Strongly agree
			Agree
			Neutral
Q8		I know that unprotected sexual contact can increase the risk of hepatitis B infection.	Strongly agree
			Agree
			Neutral
Q9		I know that hepatitis B can be passed from an infected mother to her child at birth.	Strongly agree
			Agree
			Neutral
Q10		I know that casual contact, like shaking hands, does not spread hepatitis B.	Strongly agree
			Agree
			Neutral
			Disagree
Q11		I know that the hepatitis B vaccine is safe and has few side effects.	Strongly agree
			Agree
			Neutral
			Disagree
Q12		I am aware that the hepatitis B vaccine is effective in preventing infection.	Strongly agree
			Agree
			Neutral
			Disagree
Q13		I understand that the vaccine offers long-term protection against hepatitis B.	Strongly agree
			Agree
			Neutral
			Disagree
Q14		I believe that healthcare professionals should be vaccinated against hepatitis B.	Strongly agree
			Agree
			Neutral
			Disagree
			Strongly disagree
Q15		I know that completing the full vaccination series is necessary for effective immunity.	Strongly agree
			Agree
			Neutral
Q16		I am aware of the recommended hepatitis B vaccination schedule.	Strongly agree
			Agree
			Neutral
			Disagree
			Strongly disagree
Q17		I am informed that booster doses may be recommended for high-risk individuals.	Strongly agree
			Agree
			Neutral
			Disagree
			Strongly disagree
Q18		I believe that all healthcare workers should check their hepatitis B immunity status regularly.	Strongly agree
			Agree
			Neutral
Q19		I understand that hepatitis B vaccination is the key to reducing the virus spread in healthcare settings.	Strongly agree
			Agree
			Neutral
Vaccination status			
Q20	Have you received the hepatitis B vaccine?		Yes
			No
			Not sure
	If yes, have you completed the full hepatitis B vaccination series (3 doses)?		Yes, completed all doses
			No, received only one or two doses
	If not completed, reasons for not completing the series or not taking the vaccine		Difficulty accessing the vaccine
			Fear of side effects
			Perception that the vaccine is unnecessary
			Lack of awareness about the full vaccination series
			Others (specify)
	When was the last dose of the hepatitis B vaccine received?		Within the last 6 months
			6 months to 1 year ago
			More than 1 year ago
			Not applicable
	Have you ever checked your immunity status (anti-HBsAb levels) for hepatitis B?		Yes
			No
			Not sure
